# Predicting the Long-Term Course of Asthma in Wheezing Infants Is Still a Challenge

**DOI:** 10.5402/2011/493624

**Published:** 2011-07-27

**Authors:** Flore Amat, Amandine Vial, Bruno Pereira, Isabelle Petit, André Labbe, Jocelyne Just

**Affiliations:** ^1^Asthma and Allergies Centre, Armand-Trousseau Children Hospital, University Pierre and Marie Curie-Paris 6, Paris, France; ^2^Biostatistics Unit, University Hospital of Clermont-Ferrand, Clermont-Ferrand, France; ^3^Center of Clinical Investigations, Faculty of Medicine, University of Clermont-Ferrand, Clermont-Ferrand, France; ^4^Airborne Allergies in Infants Unit, Department of Pediatrics, CHU-Estaing/University Teaching Hospital of Clermont-Ferrand, Clermont-Ferrand, France

## Abstract

*Background*. In recurrent wheezing infants, it is important to identify those likely to remain asthmatic in order to propose appropriate long-term management. *Objective*. To establish predictive factors for persistent asthma at adolescence in a population of recurrent wheezing infants. *Methods*. Retrospective study of 227 infants. Inclusion criteria were age under 36 months, a history of at least three wheezing episodes assessed via a doctor-led ISAAC questionnaire and a standardized allergy testing programme. At 13 years, active asthma was assessed by questionnaire. *Results*. Risk factors for asthma persisting into adolescence were allergic sensitization to multiple airborne allergens (OR 4.6, CI-95% (1.9–11.2) *P* = 0.001), initial atopic dermatitis (OR 3.4, CI-95% (1.9–6.3) *P* < 0.001), severe recurrent wheezing (OR 2.3, CI-95% (1.3–4.2) *P* = 0.007), and hypereosinophilia ≥470/mm^3^ (OR 2.2, CI-95% (1.07–4.7) *P* = 0.033). *Conclusion*. While it is still difficult to predict the long-term course of asthma, atopy remains the major risk factor for persistent asthma.

## 1. Introduction

The natural history of asthma is still poorly understood [1]. Diagnosis in infants often proves difficult treatment, although well-codified, poses complex practical issues at such an early age [2]. Early childhood wheezing disorders follow different time courses, probably corresponding to different endophenotypes [1, 3, 4]. Thus, wheezing phenotype is assessed retrospectively, making it impossible to integrate phenotype-specific features into asthma management policy for infants under 36 months of age [1, 5]. However, it is essential to target the population of wheezing infants likely to remain long-term active asthmatics in order to deliver appropriate adapted treatment and followup. Evidence from neonatal cohort studies suggests early-onset persistent asthma has the worst long-term prognosis [3], because of a faster decline in respiratory function during adolescence [6–8]. These studies were focused on the general population. Asthma predictive scores established in these populations are not necessarily transposable to the most severe cases of asthma followed by specialists in tertiary care [9]. For these reasons, we performed a study of a cohort of wheezing infants in a tertiary care centre to determine long-term prognosis and to identify risk factors for asthma at adolescence. 

## 2. Materials and Methods

### 2.1. Type of Study

This retrospective study was performed in two tertiary care centres: the Asthma and Allergies Centre, Armand-Trousseau Hospital, Paris (France), and the Centre for Airborne Allergies in Infants, CHU-Estaing (University Teaching Hospital), Clermont-Ferrand (France).

### 2.2. Selection Criteria and Methods

We recruited infants aged under 36 months who had a history of at least three wheezing episodes and who had been assessed for respiratory wheezing disease via a doctor-led ISAAC questionnaire [10, 11] and a standardized allergy testing programme. Infants born premature or presenting other causes of chronic airway obstruction (e.g., cystic fibrosis or bronchopulmonary dysplasia) were excluded from the study. 

The patients were contacted at the age of 13 years by a doctor, who used the ISAAC questionnaire in a telephone interview to assess the pattern of active asthma symptoms over the preceding 12 months. 

### 2.3. Predictive Parameters Measured on Infants

The predictive parameters measured and collated were (i) demographic characteristics: age, gender; (ii) parental documented history, when available, of doctor-diagnosed asthma and infant's personal history of atopic dermatitis; (iii) severe recurrent wheezing indicated by at least two previous hospital admissions due to exacerbation; (iv) biological markers of atopy: eosinophilia (hypereosinophilia was defined as an absolute eosinophil count of ≥470/mm^3^) [12], total serum IgE (total serum IgE was defined as high at ≥45 IU/mL) and allergen sensitization, as defined by the presence of allergen-specific IgE ≥0.35 kU/L (Cap System; Pharmacia & UpJohn, Saint-Quentin-en-Yvelines, France) to at least one of the common aeroallergens (dust mites, cat or dog dander, seed plant, or birch pollen) or one of the common food allergens (cow's milk, eggs, peanuts, wheat, soybean, fish). Allergen sensitization was single or multiple (≥2 specific food allergen-positive IgE or ≥2 specific airborne allergen-positive IgE). Allergen polysensitization was defined as ≥2 allergen-specific IgE, regardless of allergen class. Positivity of the modified Asthma Predictive Index (API) was assessed for each infant. 

### 2.4. Parameters Assessed at Adolescence

Persistent active asthma in the previous 12 months was defined as the presence of asthma symptoms and/or antiasthma medication taken during that period. 

### 2.5. Ethics

The study was performed in accordance with French and international best practices for epidemiological research. Consent forms were collected from the parents for every questionnaire. The Saint-Antoine Hospital (Paris) institutional review board for biomedical research did not require approval from an ethics committee, since the study protocol did not feature any investigations other than those routinely used in the day-to-day management of the patient. Data analysis was managed in such a way as to protect confidentiality as stipulated in the French *Commission Nationale d'Informatique et Libertés* data privacy law.

### 2.6. Statistical Analysis

Statistical analysis was performed using STATA v11 for Windows (StataCorp, Tex, USA). Continuous quantitative variables were expressed as means ± standard deviation and qualitative variables as numbers and percentages. The dependent variable studied was active asthma symptoms at age of 13. 

Under univariate analysis, continuous variables from the asthmatic *versus* nonasthmatic groups were compared using either a Student's *t*-test or Kruskal-Wallis test (for non-Gaussian data). Categorical variables were compared by chi-squared test or, if the conditions were unsuitable, Fischer's exact test. Statistical significance was set at *P* < 0.05. 

Predictive variables for persistence of asthma at age 13 after univariate analysis (*P* < 0.05) were introduced into a multivariate logistic regression model, allowing for correlation issues. A step-down procedure was used to establish which of the predictive variables were independent prognostic factors for persistent asthma at 13 years by calculating odds ratios and their confidence intervals. Sensitivity, specificity, and positive and negative predictive values were calculated and the receiver operating characteristic (ROC) curve was used for modified API.

## 3. Results

The two centres recruited a combined total of 541 patients, for 436 of whom fully workable datasets were compiled. We were able to recontact 227 of the 436 at age 13, who then formed the final study population. Of the remaining patients, 206 were registered as lost-to-followup (no valid address at call-up or three or four calls left without a reply) and 3 responded to call-up but declined to complete the questionnaire. The age of children lost-to-followup was significatively lower at initial diagnosis than the average age of recruits (22 ± 8,6 months, *P* < 0.001), and the children had a significantly lower incidence of atopic dermatitis (*P* < 0.001). There were no statistically significant differences in terms of initial allergen sensitization (particularly sensitization to multiple airborne allergen triggers), hypereosinophilia, family history of asthma, gender, and initial severity assessment based on hospital admission history.

### 3.1. Descriptive Analysis of Infants and Adolescents at Follow-Up End Point

Average age at the intake assessment was 26 months (SD: 8.8). Boys accounted for 68.1% of cases (*n* = 297). During infancy 96 patients (42, 2%) had had severe recurrent wheezing. Average age at the follow-up end point was 13.1 years (SD: 0.9), and over the previous 12-month period, 121 patients (55.8%) had had active asthma. 

### 3.2. Predictive Parameters of Asthma Persistence at Follow-Up End Point (Univariate Analysis)

The factors significantly associated with active asthma at the follow-up end point were presence of initial atopic dermatitis (*P* < 0.001), sensitization to multiple aeroallergens (*P* < 0.001) and to multiple food allergens (*P* = 0.02), allergen polysensitization (*P* < 0.001), hypereosinophilia (*P* = 0.001), high total serum IgE (*P* = 0.002), and severe recurrent wheezing (*P* = 0.004). In contrast, neither infant age nor infant gender was predictive of persistently-active asthma at the follow-up end point.  Table 1 gives the main results of the univariate analysis. 

### 3.3. Predictive Parameters of Asthma Persistence at Follow-up End Point (Multivariate Analysis)

The most relevant risk factors for persistent asthma were:

- sensitization to multiple aeroallergen (OR 4.6, CI-95% (1.9–11.2) *P* = 0.001),
- previous history of atopic dermatitis (OR 3.4, CI-95% (1.9–6.3) *P* < 0.001),
- initial severity (OR 2.3, CI-95% (1.3–4.2) *P* = 0.007),
- hypereosinophilia ≥470/mm^3^ (OR 2.2, CI-95% (1.07–4.7) *P* = 0.033).

Finally, of the asthmatic infants presenting all these risk factors, 95.6% remained active asthmatics in adolescence, with 81% of the cases being classifiable as mild to severe persistent.

### 3.4. Assessing Results of the Modified Asthma Predictive Index in the Study Population

Sensitivity, specificity, negative and positive negative values, and ROC Curve Area are given in Table 2. Modified API was able to correctly classify 68.7% of recurrent wheezing infants for risk of active asthma in adolescence.

## 4. Discussion

The main advantage of this study is that it was performed bicentrically, with cohorts of patients submitted to the same management procedure for both the questionnaire and the allergy testing programme. However, our findings have some limitations because of the retrospective nature of the study and the percentage of cases lost to followup (52.2%). This percentage is high but needs to be seen in the perspective of the length of the follow-up period, which ran to over 10 years. Other long-term follow-up studies have reported similar findings [13, 14]. The main characteristics of the lost-to-followup population differ from those of the final population by a lower rate of atopic dermatitis and a younger age at diagnosis, but allergen sensitization and initial severity were strictly the same. The lower incidence of atopic dermatitis in the lost cases is a bias for our result.

Unfortunately, we have no data on respiratory function parameters to corroborate persistently active asthma in the previous 12 months [5, 15] and it is known that asthma symptoms are frequently underestimated by patients and their family [16, 17]. Another potential biasing factor in this study is the lack of data on environmental factors (intercurrent viral infections, passive smoking) that may play a role in the persistence of symptoms in asthmatic children [18–20]. 

At the age of 13, 121 patients (55.8%) have had active asthma over the previous 12-month period. In an earlier study of the Paris-based cohort, asthma symptoms were observed in only 1/3 of the children at age of 6 yrs [12]; the time course of asthma tends to show fluctuating patterns over the patient's lifetime. Studies demonstrate that periods of remission may only be transient [21] and that periods of relapse are common [13]. 

The risk factors associated with persistent childhood asthma symptoms were consistent with those in the literature: multiple sensitization to aeroallergens in recurrent wheezing infants as a major risk factor for active asthma in adolescence [22–25], atopic dermatitis as an asthma-specific vulnerability-marker phenotype [3, 23, 26–28], hypereosinophilia [12, 29–31], and initial-intake severity [4, 6, 7], which indicate a probable genetic predisposition associated with specific vulnerability to environmental factors [32, 33]. 

Several authors had tried to establish scores to predict the long-term persistence of asthma. However, these scores do not have high statistical predictive power, especially when used in cohorts of wheezing infants instead of general-population infants. The most widely used score, the modified asthma predictive index (API), offers the benefit of being easy to use, but sensitivity, specificity, and positive predictive value are quite low [9], as demonstrated in our study. Negative predictive value is higher [29], but this value has little effect in term of clinical disease management. 

## 5. Conclusion

Predicting the long-term course of asthma is still difficult, although atopy is confirmed as a major predictive risk factor. Predictive scores are unfortunately not reliable enough performances to have a real value at an individual level. Early identification of clinical and biological signs of atopy is essential to target the population of wheezing infants who will need specialized long-term followup and who would greatly benefit from specific treatment.

## Figures and Tables

**Table 1:** Predictive parameters of asthma persistence at follow-up end point (univariate analysis).

	Persistent	Remission	*P* value
Atopic dermatitis	85 (70.3%)	39 (36.8%)	<0.001
Sensitization to ≥2 food allergens	15 (12.4%)	4 (3.7%)	0.02
Sensitization to ≥2 airborne allergens	40 (33.1%)	7 (6.6%)	<0.001
Allergen polysensitization	47 (38.8%)	12 (11.3%)	<0.001
Total serum IgE >45 IU/mL	59 (48.8%)	30 (28.3%)	0.002
Blood eosinophil count ≥470/mm^3^	39 (32.2%)	15 (14.2%)	0.001
Mother with asthma	27 (22.5%)	20 (19.1%)	0.52
Father with asthma	28 (23.3%)	19 (18.6%)	0.33
Initial severity	62 (51.2%)	34 (32.1%)	0.004

Allergen sensitization: presence of allergen-specific IgE ≥0.35 kU/L to at least one of the common aeroallergens (dust mites, cat or dog dander, seed plant, or birch pollen) or one of the common food allergens (cow's milk, eggs, peanuts, wheat, soybean, and fish). Allergen sensitization was single or multiple (≥2 specific food allergen-positive IgE or ≥2 specific airborne allergen-positive IgE). Allergen polysensitization was defined as ≥2 allergen-specific IgE, regardless of allergen class. Initial severity: two previous hospital admissions due to exacerbation. Hypereosinophilia: absolute eosinophil count of ≥470/ mm^3^.

**Table 2:** Assessment of the ability of modified API to predict time course development of asthma in adolescence in the study population.

Asthma at the follow-up end point	Sensitivity % (CI-95%)	Specificity % (CI-95%)	PPV %(CI-95%)	NPV %(CI-95%)	Area under the ROC curve	Correctly predicted, %
Positive API	87 (79–92)	37 (28–47)	61 (53–68)	71 (57–82)	0.62	68.7

PPV: positive predictive value; NPV: negative predictive value.

ROC: receiver operating characteristic curve.

API: asthma predictive index.
